# Loneliness and Purpose in Life Are Important Predictors for Future Care Planning

**DOI:** 10.1093/geroni/igaa057.1024

**Published:** 2020-12-16

**Authors:** Wingyun Mak, Silvia Sörensen

**Affiliations:** 1 The New Jewish Home, New York, New York, United States; 2 University of Rochester, Rochester, New York, United States

## Abstract

Experiencing purpose and social connection in later life is associated with better quality of life, better cognition, less morbidity, and lower risk of mortality. People who experience less purpose in life are more likely to report loneliness (Neville et al., 2018), and those with vision impairment are at greater risk for loneliness than the general population (Brunes et al., 2019). Planning for future care may be one way to enhance late life outcomes, but it is unclear how loneliness and purpose in life are related to planning behaviors in older people with vision loss. Using a sample of 200 older adults who were diagnosed with macular degeneration, this study explored the association of loneliness and purpose in life on future care planning variables after controlling for basic health and demographic variables. Hierarchical regressions showed that 1) people who are lonelier (β=.26, p<.05) but report greater purpose in life (β=.19, p<.05) are more aware of future care needs (ΔR2= .14, p<.001); 2) people who are lonelier (β=-.16, p<.05) but report greater purpose in life (β=-.46, p<.001) are less avoidant of planning (ΔR2= .14, p<.001); and 3) people who report greater purpose in life are more likely to gather information (β=.24, p<.05; ΔR2= .04, p<.05) and establish concrete plans related to planning for future care (β=.25, p<.05; ΔR2= .06, p<.001). These results suggest that having purpose in later life may boost planning behaviors, while those who are lonely may need help translating their awareness of future care needs into planning behaviors.

